# Visualization of membrane protein crystals in lipid cubic phase using X-ray imaging

**DOI:** 10.1107/S0907444913011359

**Published:** 2013-06-15

**Authors:** Anna J. Warren, Wes Armour, Danny Axford, Mark Basham, Thomas Connolley, David R. Hall, Sam Horrell, Katherine E. McAuley, Vitaliy Mykhaylyk, Armin Wagner, Gwyndaf Evans

**Affiliations:** a Diamond Light Source, Harwell Science and Innovation Campus, Didcot OX11 0DE, England; b Oxford e-Research Centre, 7 Keble Road, Oxford OX1 3QG, England; c University of Liverpool, Liverpool L69 3BX, England

**Keywords:** membrane proteins, lipid cubic phase, microradiography, microtomography

## Abstract

The focus in macromolecular crystallography is moving towards even more challenging target proteins that often crystallize on much smaller scales and are frequently mounted in opaque or highly refractive materials. It is therefore essential that X-ray beamline technology develops in parallel to accommodate such difficult samples. In this paper, the use of X-ray microradiography and microtomography is reported as a tool for crystal visualization, location and characterization on the macromolecular crystallography beamlines at the Diamond Light Source. The technique is particularly useful for microcrystals and for crystals mounted in opaque materials such as lipid cubic phase. X-ray diffraction raster scanning can be used in combination with radiography to allow informed decision-making at the beamline prior to diffraction data collection. It is demonstrated that the X-ray dose required for a full tomography measurement is similar to that for a diffraction grid-scan, but for sample location and shape estimation alone just a few radiographic projections may be required.

## Abstract 2

A comparison of X-ray diffraction and radiographic techniques for the location and characterization of protein crystals is demonstrated on membrane protein crystals mounted within lipid cubic phase material.

## 1. Introduction

As macromolecular crystallography (MX) beamlines become brighter and diffraction data are collected from ever-smaller crystals, there are a number of practical difficulties that have emerged that require attention. Data are being collected from ever more challenging crystal samples that are often in­homogeneous, weakly diffracting and small, and in many ways the determination of the structure of challenging targets, such as membrane proteins and viruses, is more achievable than ever (Lyons *et al.*, 2012 ▶; Shimamura *et al.*, 2011 ▶; Doré *et al.*, 2011 ▶; Wang *et al.*, 2012 ▶). However, many of these structures require the characterization of hundreds of crystals and make the process of data collection laborious and time-consuming. The bottlenecks in data collection are related to sample visualization, location and characterization, especially where tens or hundreds of crystals per structure must be measured.

In recent years, microfocus macromolecular beamlines have become increasingly used at synchrotron sources (Evans, Axford, Waterman *et al.*, 2011 ▶) owing to their ability to probe very small crystals or more perfect regions of larger crystals. Careful matching of the beam size to the crystal size increases the signal-to-noise ratio and leads to better quality diffraction data (Evans, Axford & Owen, 2011 ▶). A prerequisite of such an approach is of course knowledge of the crystal shape and size, which provides a further incentive for pre-characterization of the crystal sample prior to measuring diffraction data.

A key complication, especially in the field of membrane proteins, is the trend for crystallization in lipid cubic phase, which causes the crystals to become optically invisible once mounted (Caffrey, 2000 ▶) owing to the opacity of cubic phase material. This makes both the location of the crystals and the determination of their size and shape very challenging using visible light. Furthermore, crystals may be too small to view easily once mounted within a larger loop or mesh, or the crystals may be mounted or embedded in a refractive material which causes the sample to appear shifted relative to its actual location (Axford *et al.*, 2012 ▶; Wagner *et al.*, 2009 ▶). In all of these cases centring the crystals is problematic and has led to the development of raster- or grid-scanning methods that are routinely adopted on many microfocus beamlines (Aishima *et al.*, 2010 ▶; Bowler *et al.*, 2010 ▶; Cherezov *et al.*, 2009 ▶). The alignment of the crystals prior to the diffraction experiment is a key step in the experimental procedure in order to obtain optimal results.

There are numerous optical and X-ray techniques that are being developed or are already available to help with this visualization. One such technique uses second-order nonlinear optical imaging of chiral crystals (SONICC; Kissick *et al.*, 2010 ▶). This technique is good at discriminating between protein and salt crystals and can be used with microcrystals, but to date has only been used to screen crystallization trays prior to mounting the sample for X-ray diffraction measurements, although such equipment might be integrated into beamline visualization systems.

The use of X-ray microtomography in MX for determining the details of crystal shapes for use in absorption corrections has already been successfully demonstrated (Brockhauser *et al.*, 2008 ▶). In this paper, the use of X-ray tomography and radiography is explored for application to the location and characterization of crystals prior to data collection and their use is compared with the existing diffraction rastering methods. The relative effects of radiation damage in the two methods are evaluated and further reductions in dose using radiography and visual hull reconstructions are considered.

## 2. Methods

### 2.1. X-ray tomography and radiography apparatus

**Figure 1:**
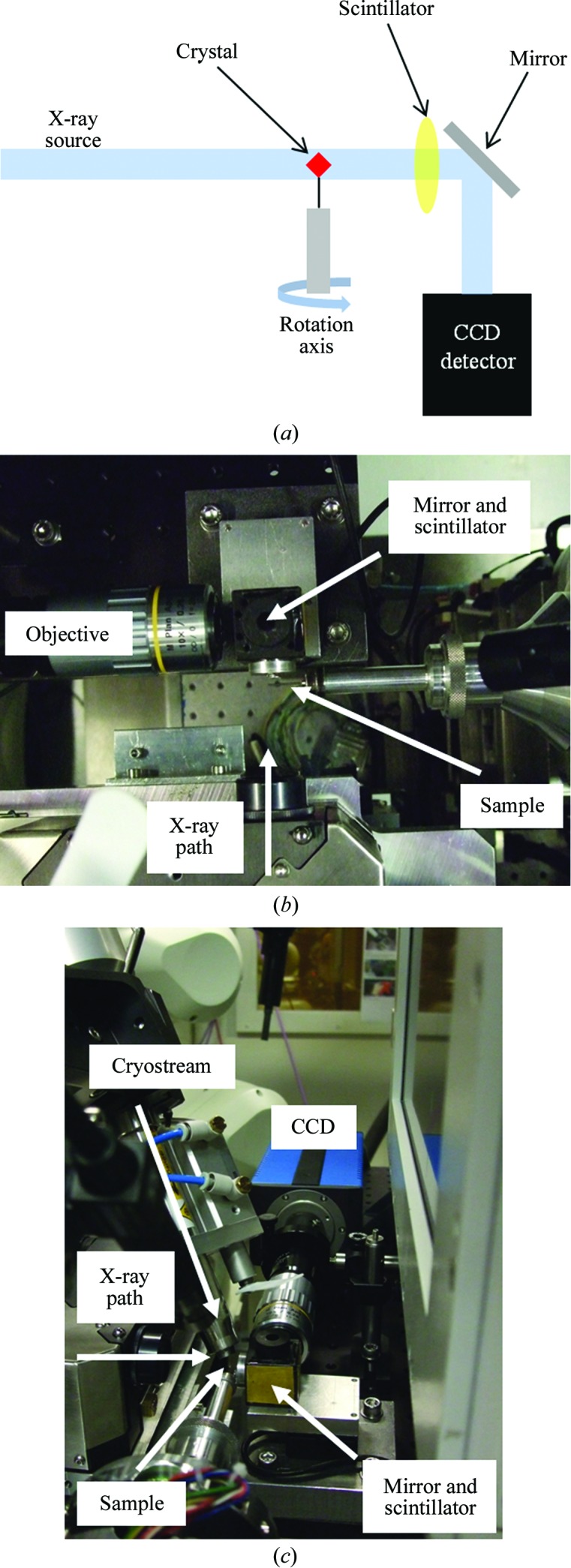
The experimental setup for X-ray tomography and radiography, showing (*a*) a schematic representation demonstrating the basic principles of X-­ray microtomography and radiography, (*b*) the setup on beamline I04 at Diamond viewed from above and (*c*) the same setup viewed from the side, showing the installation of the equipment below the detector support.

The basic principle of an X-ray radiography or tomography measurement is illustrated in Fig. 1 ▶(*a*). A large, stable and uniform X-ray beam is projected onto the sample, and contrast in the absorption between the sample, mount, mother liquor and air is observed on the scintillator. The resulting image is collected on the camera. These images can be used in conjunction with flat-field images, measured without a sample in the beam, to obtain a quantitatively accurate radiograph of the sample. The crystal may then be rotated and many images collected at successive angular increments. With a sufficient number of images covering 180° of sample rotation (for the parallel beam case), a computer algorithm can be used to generate a three-dimensional tomographic reconstruction of the sample. This can provide detailed information about the sample such as the orientation of crystal facets and the nature of defects within its volume, as well as information about the location of the crystal within the loop and liquor.

The X-ray microtomography equipment was installed below the main detector support on beamlines I04 and I03 at the Diamond Light Source (Figs. 1 ▶ *b* and 1 ▶ *c*) and allowed con­venient interchange between tomography/radiography and diffraction experiments. The apparatus consists of a YAG:Ce scintillator (CRYTUR; http://www.crytur.cz) mounted within a small holder together with a 45° mirror that reflects the image onto a PCO 1600 CCD detector *via* a ×10 magnification objective. The setup could be translated along the beam direction to bring the scintillator to within 5 mm of the sample (ultimately limited by the width of the 100 K gaseous N_2_ stream used to cool the sample). The scintillator and mirror were mounted on a translation stage, permitting the image on the scintillator to be brought into focus on the camera.

The sample-rotation axis on diffraction beamlines is aligned orthogonal to the X-ray beam, as this is the preferred orientation for the diffraction data-analysis software. This alignment is also important for the tomographic reconstruction software. Owing to the beamlines already being well aligned for diffraction experiments, this was deemed to be sufficient for the corresponding tomography/radiography experiments.

The scintillator was a 25 µm yttrium aluminium garnet (YAG; Y_3_Al_5_O_12_) single crystal activated with cerium. For the radiography experiments, an X-ray energy of 5 keV (2.48 Å) was used. Radiography measurements were carried out on beamline I04 in the absence of any focusing optics, producing a large and uniform beam of approximately 700 × 500 µm (horizontal × vertical) in size. On beamline I03, vertical and horizontal focusing mirrors were defocused in order to produce a beam with dimensions large enough to completely illuminate a pair of defining slits used to set the beam size such that the complete sample was bathed in the beam. Data collection on the PCO CCD detector was triggered by a TTL signal from the sample-rotation stage controller, such that images were collected one at a time with the experimental fast shutter closing between every image. The resolution of the whole experimental setup was measured using the JIMA test pattern RT RC-02B (JIMA; http://www.jima.jp/) capable of measuring resolution down to 0.4 µm. The resolution for this setup was determined to be 2 µm.

### 2.2. Sample crystals

Membrane protein crystals grown and mounted in lipidic cubic phase (LCP) are particularly difficult to visualize using visible microscopes. Two samples prepared in LCP were used in these imaging studies: the A_2A_ adenosine G-protein coupled receptor (A_2A_) and bacteriorhodopsin.

Crystals of A_2A_ were prepared and crystallized as reported previously (Jaakola *et al.*, 2008 ▶). A nylon loop was used to mount two of these crystals (80 × 60 × 60 and 50 × 40 × 30 µm) before flash-cooling them in liquid nitrogen ready for data collection.

Bacteriorhodopsin crystals were grown in lipidic cubic phase as previously reported (Nollert, 2004 ▶). A crystal of approximate dimensions 20 × 5 × 5 µm was mounted in a nylon loop and flash-cooled in liquid nitrogen.

Crystals of copper nitrite reductase (CuNiR) were used to study the effects of radiation damage. They were prepared as previously reported (Antonyuk *et al.*, 2005 ▶); however, the samples were isolated from transformed *Escherichia coli* (BL21) instead of *Achromobacter cycloclastes*. A suitable crystal (50 × 50 × 50 µm) was selected, mounted in a nylon loop and soaked in sodium malonate before plunge-cooling in liquid nitrogen.

### 2.3. Data collection

#### 2.3.1. A_2A_

The previously cooled crystals of the membrane protein A_2A_ were manually mounted on the goniometer on beamline I04 at 100 K in the open flow of the nitrogen-gas cryostream. It was not possible to mount the sample using the robotic sample changer owing to the positioning of the tomography apparatus: future designs will accommodate use of the sample changer and allow routine automated operation. The sample was centred and 360° of visual images at increments of 1° were taken using an on-axis video (OAV) microscope integrated into the beamline end station. These were taken to act as a comparison with the corresponding X-ray projection images. The scintillator was positioned approximately 5 mm from the sample position and the X-ray energy was set to 5 keV (2.45 Å) with 100% transmission of the beam, resulting in a total flux of 3 × 10^9^ photons s^−1^ measured on a calibrated silicon PIN diode. An exposure time of 500 ms per image was used. An initial projection was collected with the sample in the beam path; the sample was then translated fully out of the beam so that a flat-field image could be recorded. This was followed by 18 projections of the sample at angular increments of 1° and then by another flat-field image. This procedure was repeated ten times until a total angular range of 180° had been recorded with a flat-field image measured every 18°. It was necessary to use long exposure times of 500 ms because residual beam instabilities were observed on faster timescales; these were averaged out at 500 ms. The series of flat-field images was used to correct for slower beam movements observed over the duration of the experiment. No dark images were measured, as it had been determined that dark-current correction had little effect on the data quality.

#### 2.3.2. Bacteriorhodopsin

The bacteriorhodopsin sample was studied using both tomography and diffraction grid-scanning. The cooled crystals were manually mounted on the goniometer on beamline I03, with the cryostream set to 100 K. A coarse grid-scan was first measured using an energy of 12.7 keV (0.98 Å) and a beam size of approximately 40 × 40 µm for the scan; the corresponding box size for the grid was also set to this value, with approximately fivefold lower flux than a standard diffraction image. An 8 × 5 grid was recorded to cover the whole loop. Data were analysed using *DISTL* (Zhang *et al.*, 2006 ▶) and the best Bragg candidate score was used to produce a contour map to overlay onto the sample image. The contour map was produced by interpolating between the scores calculated from *DISTL* to produce a plot which is easier to interpret. X-ray radiography measurements were then performed on the same sample for comparison with results from the grid-scan. The same strategy and X-ray energy were used for the collection of the bacteriorhodopsin tomography data set as for A_2A_ in the previous section.

#### 2.3.3. Copper nitrite reductase

Crystals of CuNiR were used to study the radiation-dose effect of X-ray tomography/radiography on the samples. The previously cooled crystals were manually mounted and diffraction data were collected on beamline I04 using 9.0 keV (1.38 Å) X-rays with a beam size of 150 × 150 µm using compound refractive lenses. The data collections consisted of 1° oscillations, 3 s exposures and 180 images with 100% transmission of the beam (equating to ∼5 × 10^10^ photons s^−1^). After this, six tomography data sets were recorded following the same procedures as used for A_2A_ and bacteriorhodopsin. Finally, a second diffraction data collection was performed using identical parameters so that the relative contribution to radiation damage of the tomography data collection could be assessed. The crystal remained in the same orientation in both the diffraction and tomography experiments so that any changes could be directly related to the dose from the tomography experiments. The data were analysed using *XDS* (Kabsch, 1993 ▶) and *SCALA* (Evans, 2006 ▶) for the ‘before’ and ‘after’ data collections.

### 2.4. Radiographs and reconstructions

The ten flat-field images that were recorded for each data collection were interpolated to produce a flat-field image that corresponded to every individual data image in that series. Each sample projection was then divided by its own interpolated flat field to produce a series of corrected radiograph images. All tomographic reconstructions reported here were produced with *TomoJ* within the *ImageJ* package (Abramoff *et al.*, 2004 ▶; Messaoudii *et al.*, 2007 ▶) and used the iterative algorithm *SIRT* (*Simultaneous Iterative Reconstruction Technique*; Kak & Slaney, 1988 ▶).

## 3. Results

### 3.1. Crystal location

**Figure 2:**
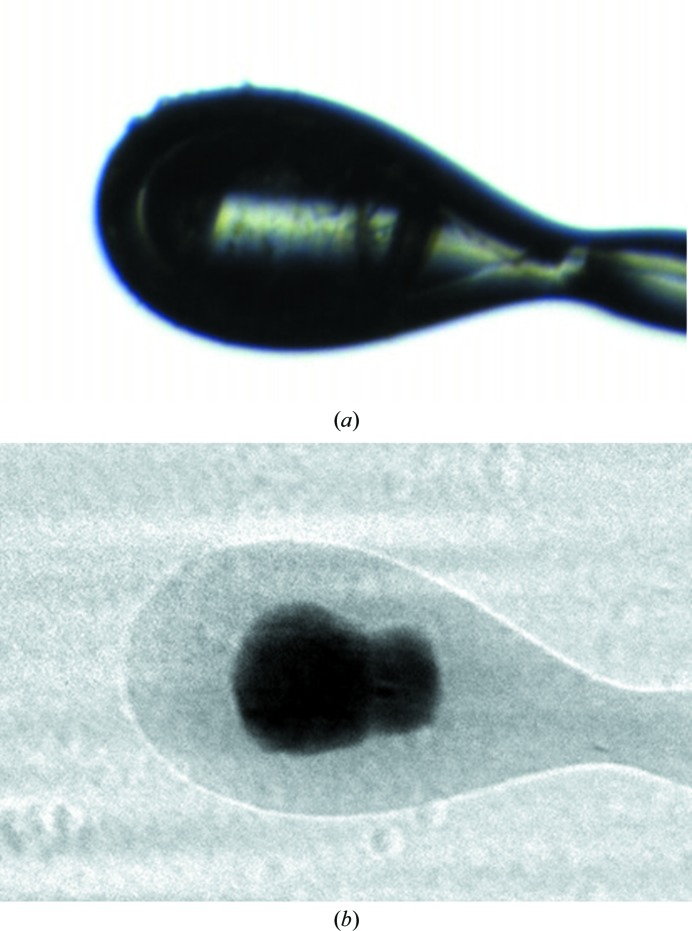
(*a*) View of membrane protein crystals of the human A_2A_ adenosine G-­protein coupled receptor in lipid cubic phase mounted on a nylon loop using a visible microscope; (*b*) the same orientation of the sample viewed as a radiograph. It is unclear in (*a*) where the crystals are located, while after X-ray imaging (*b*) it is evident that two crystals are located within the loop.

Fig. 2 ▶ shows the view of the A_2A_ sample as seen on the OAV microscope and also a flat-field-corrected radiograph in the same orientation. The absorption contrast ratio between the crystal and the LCP material is 2.2 and there appear to be two crystals visible in the projection. By comparison, in the visible-light image it is difficult to draw any solid conclusions about the size, location or shape of the crystal(s). This illustrates how radiography can provide more detailed and faithful information about crystals within optically opaque material. In this instance, an initial projection image could enable better decision-making about the choice of beam size for subsequent diffraction experiments.

**Figure 3:**
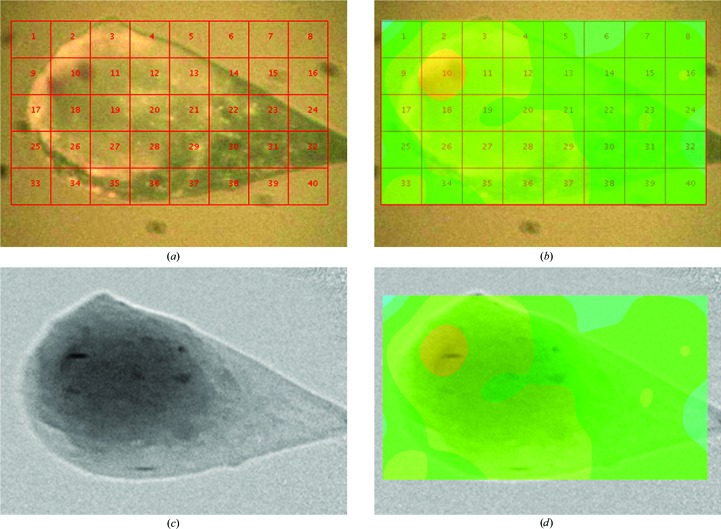
View of the membrane protein crystal bacteriorhodopsin in lipid cubic phase mounted on a nylon loop (*a*) with the grid-scan set up for data collection, (*b*) the results of the good Bragg candidates from the grid-scan calculated using *DISTL* (Zhang *et al.*, 2006 ▶) overlaid on the sample image, (*c*) the same orientation of the sample viewed as a radiograph and (*d*) the grid-scan results overlaid on the radiograph. The grid-scan and the radiograph both demonstrate how they can be used for determination of the sample location and both results map well, as can be seen in (*d*). The radiograph in (*c*) provides more information about the crystal size and shape which can then be used to allow better decision-making at the beamline for the diffraction experiment.

The results of the bacteriorhodopsin experiments described in §[Sec sec2.3.2]2.3.2 from both imaging and the diffraction grid-scan are shown in Fig. 3 ▶. The mounted crystals grown in LCP were difficult to optically centre using the OAV microscope and it was not clear exactly where the crystals were located. Grid-scanning and radiography were therefore used. Fig. 3 ▶(*a*) shows the OAV image of the sample with the grid-scan overlaid and Fig. 3 ▶(*b*) shows the good Bragg candidates from *DISTL* (Zhang *et al.*, 2006 ▶) plotted as a contour map. The best position from the scan is grid 10.

The same sample was then studied using X-ray radiography to see how the results matched those observed in the grid-scan. The sample projection with the loop in the same orientation as that of the grid-scan was divided by the flat-field image to give the radiograph shown in Fig. 3 ▶(*c*). The absorption contrast ratio between the bacteriorhodopsin crystals and LCP is 1.21, and it is therefore apparent where several small needle crystals are located within the loop. The projection and grid-scan results were overlaid manually (a scale factor and rotation around the beam axis were applied) to demonstrate the correlation of the two techniques. Indeed, one of the most significant needle-like shapes in the radiography measurement corresponds to the strongest diffraction region of the sample as assessed by diffraction grid-scanning.

The grid-scan indicates position 10 as a good diffracting position; however, there was no measured diffraction from other areas of the sample, even though the projection indicates the presence of several other microcrystallites. The grid-scan used a beam size of 40 × 40 µm, which is much larger than the crystals in the loop, increasing the background and reducing the signal-to-noise ratio from any crystal diffraction. In this case, the use of the projection image would guide a user in selecting a suitable beam size in order to optimize the measurement and to achieve the best possible result from the diffraction.

### 3.2. Comparison of diffraction and imaging techniques

The results in Fig. 3 ▶ illustrate that both radiography and grid-scanning methods can provide information about the location of a crystal in opaque material. However, there are a number of differences between the two techniques as they rely on different types of information.

Grid-scanning provides important information about the diffraction quality of crystals as well as their location. However, determining the size and shape of crystals is limited by the resolution of the grid-scan measurement and is determined, in each direction, by whichever is the larger of the beamsize and the grid step size (40 × 40 µm for the measurement shown in Fig. 3 ▶). It is important to note that grid-scanning is a two-dimensional technique and therefore obtaining three-dimensional information about the crystal shape for centring requires repeated grid-scans in other orientations. Obtaining high-resolution three-dimensional information would require the use of a microbeam scanned across the sample in multiple crystal orientations.

In the case of tomography/radiography we derive no information about the diffraction quality of a crystal, but learn much more about the crystal location, size and shape, typically down to a resolution of 2 µm (Borbély *et al.*, 2011 ▶; Brock­hauser *et al.*, 2008 ▶). This increase in resolution gives a more detailed representation of crystal morphology and is critical in determining which beam size and shape should be used in order to achieve optimal signal-to-noise in the diffraction experiment (Evans, Axford & Owen, 2011 ▶).

### 3.3. Radiation damage

To establish the relative X-ray doses delivered to a crystal sample in grid-scanning and tomographic methods, crystals of CuNiR were used to perform several sets of diffraction and tomography measurements. Standard data collections were used as opposed to grid-scanning to simplify calculation of the dose delivered to the sample, but knowledge that typical grid-scans are performed using roughly a fivefold lower flux allows a final comparison to be drawn between the two methods.

**Figure 4:**
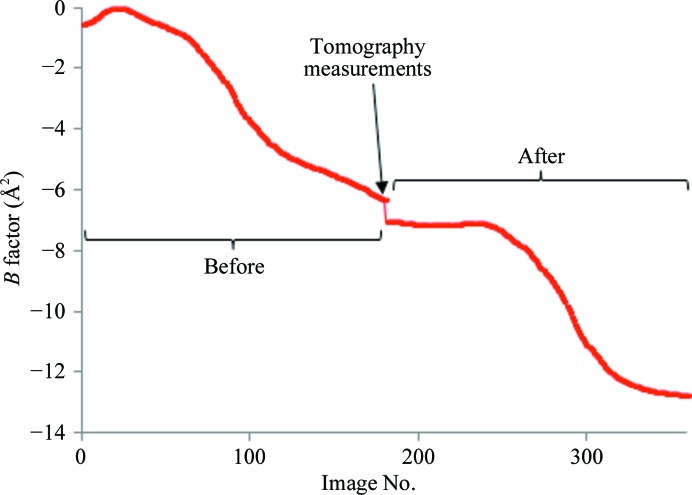
A plot of the scaling results from *SCALA* (Evans, 2006 ▶) for CuNiR plotted as image number *versus* the *B* factor. Images 0–180 are from before the imaging experiments and images 181–360 are the diffraction images after the experiments.

Diffraction data were collected as described earlier, followed by six full 180° tomographic data sets. Finally, the diffraction experiment was repeated using identical data-collection parameters. The results of data analysis for the ‘before’ and ‘after’ data collections are shown in Table 1 ▶. It is evident from the increase in the unit-cell dimension and the increase in *R*_p.i.m._ for the outer resolution shells that there is a drop-off in the data quality owing to radiation damage throughout the whole experiment. Fig. 4 ▶ shows evidence of radiation damage occurring during the ‘before’ and ‘after’ data sets and indeed shows some evidence of radiation damage owing to the tomography data collections in the form of a discontinuity in scaling *B* factor. To determine the relative impact of dose on the crystal sample two approaches have been used and are described below.

#### 3.3.1. Quantitative approach from dose on sample

*RADDOSE* (Paithankar *et al.*, 2009 ▶; Murray *et al.*, 2004 ▶) was used to quantify the relative doses delivered into the sample during tomography and diffraction. The experimental parameters for the diffraction and tomography experiments were input into *RADDOSE* and the dose for a single diffraction image and radiograph were compared. The program takes account of the crystal size, unit cell, composition and solvent content, the beam size, flux and exposure time. However, it does not account for any mother liquor that may surround the crystal. The absorbed dose per image for the diffraction experiment was found to be 5450 Gy. For an equivalent grid-scan image this would be fivefold less at 1090 Gy owing to the reduction in dose that the crystal receives during a typical grid-scan. The equivalent dose for a single radiograph was 11.2 Gy, and a corresponding three-dimensional tomography data collection would have a dose of 2016 Gy. One can see that the full three-dimensional tomographic approach is two times more damaging to the crystal than one grid-scan image. However, two-dimensional positional information equivalent to a grid-scan might be obtained with a single radiograph and this would result in ∼100-fold less dose being received by the sample.

#### 3.3.2. Empirical approach from data scaling

Of most importance to crystallographers is the impact of dose on the quality of diffraction data. Fig. 4 ▶ shows a plot of the image *B* factor from the scaling program *SCALA* (Evans, 2006 ▶) *versus* diffraction-image number for both the ‘before’ (images 1–180) and ‘after’ (images 181–360) data sets. Both the ‘before’ and the ‘after’ data sets collected the same images with the crystal in the same orientation, so the discontinuity in *B* factor between the two is owing to the dose delivered during the six tomography measurements.

An estimate of the impact of this dose can be made using Fig. 4 ▶. It is possible to see from the plot that the *B* factor reduces by ∼6 Å^2^ during both the ‘before’ and the ‘after’ diffraction data sets. Six tomography data sets, each consisting of 180 images, result in a total reduction in *B* factor of 0.7 Å^2^. Therefore, one can deduce that a single 180-frame tomography data set is equivalent in ‘damage’ to 3.5 {= [(0.7/6) × 180]/6} individual diffraction images. Conversely, one diffraction image is equivalent to ∼51 radiographs and one grid-scan image is equivalent to ∼10 radiographs (assuming a dose that is lower by a factor of five for a grid-scan image).

It should be noted that this estimate is very approximate since the *B*-factor determination from *SCALA* is itself only an approximate indicator of radiation damage and is very dependent on the scaling protocol used and the quality of the data. The conclusion, however, is that a single radiograph is ten times less damaging than a grid-scan in which the *B* factor is used as an indicator of sample damage.

The two approaches outlined compare the delivered dose during radiography and grid-scanning, which can both be used to locate the sample prior to the diffraction experiment. However, there is more than an order-of-magnitude discrepancy in the results obtained using the two methods outlined above.

The dose calculation from *RADDOSE* suggests that a single radiograph is ∼100 times less damaging than a single grid-scan exposure, whereas the *B* factor estimate suggests that this ratio is only 10. However, it is clear that if radiography were used for sample location, in which only a few projections of the sample are needed, it is potentially a lower dose measurement than equivalent grid-scans.

### 3.4. Radiographic projections and visual hull reconstructions

The experiments carried out above involved the measurement of full 180° sets of tomography data, exposing the crystal to X-rays for a relatively long period of time. However, to determine the crystal location and to obtain an idea about the basic crystal shape a smaller number of images could be required, thereby exposing the crystal to a far lower dose. The number of projections collected is already minimal for tomography, so the use of a small number of radiographic projections was investigated using visual hull reconstruction to produce an approximate but sufficient representation of the crystal size and shape.

**Figure 5:**
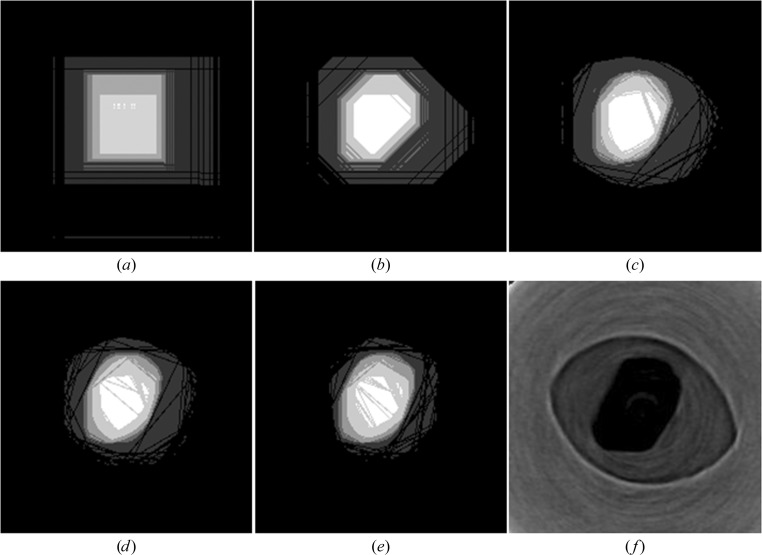
Visual hull reconstructions of the same slice through the A_2A_ crystal along the pin axis, with a varying number of projections evenly spaced over the sequence of 180 × 1° frames. (*a*) Two projections (90° apart), (*b*) four projections (45° apart), (*c*) nine projections (20° apart), (*d*) 36 projections (5° apart) and (*e*) 180 projections (all frames). The same slice through a tomographic reconstruction is also shown in (*f*), where the crystal is visible in black surrounded by LCP in the loop in dark grey. It can be clearly seen that even with a visual hull reconstruction using four projections (*b*) a good approximation to the correct shape can be made when compared with the tomographic slice (*f*).

Although not standard for visual hull reconstruction (Laurentini, 1994 ▶), transmission parallel beam radiography adapts very easily to the technique and, in circumstances where features can be identified from the radiographs directly, can provide good-quality reconstructions from very few projections. The premise is similar to tomography, apart from that where tomographic reconstruction is additive, in that each new projection adds more information to the volume and enhances the feature, the visual hull approach is subtractive: each new projection reveals regions that are not part of the object and removes these areas. As the number of projections increases the volume of the feature is steadily reduced towards a convex-hull binary representation of the object. In this case, owing to the noisy data, there are two problems associated with the approach. The first is that the original projections need to have a threshold applied in order to determine which regions need to be removed, and picking an appropriate level for this threshold is challenging. The second problem is that owing to the noise in the data, even if a good threshold is selected the result will have artefacts around the edge of the feature. To deal with these issues, we modified this approach to use multiple threshold levels to segment each frame using a coarse scale (ten evenly spaced segmentation levels) and then sum the resulting frames to make a single final reconstruction. This produces a slightly higher bit depth image (about 3 bit) with higher values towards the centre of the feature. This results in the edges of the feature being visually better defined. This simple modification of the technique can identify the general shape and location of the feature of interest, in this case the crystal. Fig. 5 ▶ illustrates that as few as four radiographic projections separated evenly over 180° can provide sufficient detail about position and shape to allow an experimental strategy to be worked out. However, in general the number of individual frames required will depend on the shape and orientation of the object as well as the level of detail desired.

## 4. Conclusion

With the move to more challenging protein targets such as membrane protein crystals embedded in LCP and the production of microcrystalline samples, it has been necessary to improve beamline visualization techniques to cope with this ever-demanding area. The method reported here uses X-­ray microradiography and tomography to image the crystals for sample location.

It has been shown that it is possible to image protein crystals on dedicated macromolecular beamlines (Brock­hauser *et al.*, 2008 ▶) and that this method can be used to locate the crystals when they are impossible to see using a visible microscope. The design of the setup allows both radiography and diffraction experiments to be run in sequence, allowing projection images to be carried out prior to diffraction data collection. It has also been demonstrated that the dose delivered during the measurement of a full tomography data set is two times more damaging than that of a diffraction grid-scan. However, this dose can be reduced by only collecting as few as four radiographs, which contain sufficient three-dimensional information to indicate where the crystals are located within the loop and their approximate shape.

From the empirical estimate of sample damage to the CuNiR example (§[Sec sec3.3.2]3.3.2), it is calculated that the dose required to locate a crystal and obtain approximate shape information using four radiographic projections would be less than half of the dose delivered during the measurement of a single grid-scan. This shows the potential of X-ray radiography as a low-dose method of locating and characterizing the morphology of very radiation-sensitive crystals prior to making any diffraction measurements. However, the relative doses determined using *RADDOSE* suggest that radiography would be a very low-dose method relative to grid-scanning.

It is unclear exactly where the discrepancy between the two methods arises, but the empirical method of relative damage assessment using *B* factors is prone to large errors, as is the calculation of dose from *RADDOSE* owing to uncertainties in the absorption data for elements. Furthermore, the variation in sensitivity to X-rays with protein type might generate variation in this behaviour for other samples. In conclusion, the results presented are to be used as indicators of the relative damage caused by both methods rather than accurate rules for planning experiments.

The radiographs from the bacteriorhodopsin microcrystals suggest that sufficient contrast between lipid and protein is observable even for crystals as thin as 5 µm. Investigations of the limits of this method for microcrystals will be the topic of further investigation, but these studies have already demonstrated that for two membrane protein crystal species, A_2A_ and bacteriorhodopsin mounted in LCP, significant absorption contrast is observed at X-ray energies of 5 keV.

Although radiographs provide a great deal of information about crystal location and size, they do not provide any insight into the quality of the diffraction. For these reasons, in the future a combination of both radiography and diffraction could be used to help to determine the experimental parameters that should be set up for the diffraction experiment.

It has been demonstrated that X-ray microradiography is a significant development towards the location and characterization of samples embedded in opaque materials. Knowledge of crystal shape and size prior to obtaining any diffraction data set or even a grid-scan should greatly improve the signal-to-noise ratio of the data, which is especially important for very challenging crystals, by permitting careful selection of the beam size and shape.

## Figures and Tables

**Table 1:** Data-collection statistics before and after the tomography experiments

	Before tomography	After tomography
Wavelength (Å)	1.380	1.380
Space group	*P*2_1_3	*P*2_1_3
Unit-cell parameters (Å, °)	*a* = *b* = *c* = 94.886, α = β = γ = 90	*a* = *b* = *c* = 95.175, α = β = γ = 90
Resolution (Å)	42.43–2.21 (2.28–2.21)	42.53–2.21 (2.28–2.21)
*R*_p.i.m._	0.032 (0.155)	0.045 (0.335)
〈*I*〉/〈σ(*I*)〉	23.3 (5.6)	18.0 (2.7)
Completeness (%)	100.0 (100.0)	100.0 (100.0)
Multiplicity	21.7 (21.8)	21.7 (21.7)
No. of reflections	315180 (26786)	317994 (27142)
No. of unique reflections	14544 (1230)	14654 (1249)
